# AEGIS, the Virtual European Genebank: Why It Is Such a Good Idea, Why It Is Not Working and How It Could Be Improved

**DOI:** 10.3390/plants10102165

**Published:** 2021-10-12

**Authors:** Theo van Hintum, Johannes M. M. Engels, Lorenzo Maggioni

**Affiliations:** 1 Centre for Genetic Resources, The Netherlands (CGN), Wageningen University & Research (WUR), P.O. Box 16, 6700AA Wageningen, The Netherlands; 2 Alliance of Bioversity International and CIAT, Via di S. Domenico, 1-00153 Rome, Italy; j.engels@cgiar.org (J.M.M.E.); l.maggioni@cgiar.org (L.M.); 3 European Cooperative Programme for Plant Genetic Resources (ECPGR), Via di S. Domenico, 1-00153 Rome, Italy

**Keywords:** PGRFA, ECPGR, virtual European genebank, AEGIS, EURISCO, genebank quality management

## Abstract

Europe is very active in terms of conserving plant genetic resources, with hundreds of genebanks and thousands of dedicated people involved. However, the resulting infrastructure is, along with being very expensive, far from efficient and not very reliable. In this opinion paper, the authors describe how this situation arose, and why the European Cooperative Programme for Plant Genetic Resources (ECPGR), the collaborative umbrella organization of the European countries involved, has not been able to improve this situation so far significantly. The principles of the decentralized virtual genebank (AEGIS) are described, and an analysis is made of the reasons for its lack of success. Possible changes for making AEGIS a success, or at least steps in the right direction, are proposed. These changes center around the creation of a system of certified genebanks with proper quality management, guaranteeing the long-term conservation of, and immediate access to the plant genetic resources conserved in it.

## 1. Introduction

Plant Genetic Resources for Food and Agriculture (PGRFA) need to be conserved and made accessible for use in crop research and plant breeding. There are various reasons for this, the most prominent being the high rate of genetic erosion of PGRFA, i.e., without conservation, the genetic resources will disappear quickly [1]. Without these genetic resources, the world will not be able to feed its fast-growing population, since crops can no longer be adapted to the drastic, changing environments (biotic and abiotic factors).

The awareness of the importance of the conservation of, and access to PGRFA started with collecting activities and the research of pioneers such as, most prominently, N.I. Vavilov and H.V. Harlan in the first half of the 20 th century. The first dedicated genebanks were established in Germany, the USA, and at the large international agriculture institutes of the CGIAR during the second half of the 20th century, and soon after, many crop research institutes upgraded their working collections into genebanks [2]. Many countries wanted to have their own national genebank or a network of genebanks located predominately in research institutes that specialized in specific crop groups. Some of the resulting genebanks are no more than poorly maintained working collections, whereas others are professionally managed long-term collections, stored in well-equipped genebanks, meeting all international genebank standards and operating on the basis of a proper quality management system.

EURISCO, the database that gathers, processes, stores and makes available information about all European genebank collections, identifies at present some 400 institutes maintaining PGRFA collections in Europe (*sensu lato*, including 43 countries; for an overview of the data included in EURISCO, see Table 1). The resulting genebank landscape is very heterogeneous, since it has grown spontaneously, without any prior plan or coordination. It could be described as chaotic, involving hundreds of institutes, thousands of people and costing huge amounts of money. But despite all the money being spent, it is not clear at all if these genebanks and other PGRFA-related institutes are adequately functioning, i.e., if they are properly maintaining the PGRFA that should be conserved for the long term and made available to users. Furthermore, it is clear that this ‘system’, based on national and institutional centers, was never designed and set up to serve collaborative conservation objectives aimed at increasing efficiency and cost-effectiveness in such a fragmented landscape as Europe. Each European genebank formulated its own goals and has been reaching these goals, to varying extents, while being largely independently from related institutions and certainly from those in other countries. However, now that this European landscape has existed for nearly 50 years, the common goal has become more prominent, as have the doubts about efficacy and efficiency.

Although it is clear that much valuable germplasm material is properly conserved in the well-functioning genebanks, it is also clear that many genebank operations are not effective at all, and certainly from an overarching European or global perspective. For example, (1) much material is duplicated in many collections, while other important material for a given crop or species might be missing in all the genebanks [4]; (2) access to the conserved materials for users, if there is access at all, is often restricted to a small group, consisting of colleagues in the institute, partners of a project or members of a restricted network. Most importantly, (3) the quality of the conservation methodologies and of the conserved material is often very low.

The change in the genetic resources paradigm—caused by the Convention on Biological Diversity (CBD) that was agreed upon in 1992—from genetic resources being a ‘heritage of mankind’ to a resource ‘under national sovereignty’ did not improve the situation [5]. Access rules dictated by national governments often did not exist (certainly not before the entrance into force of the CBD in 1993) or became stricter and/or more complex thereafter. Since then, some genebanks have needed permission from national authorities to distribute material abroad for each distribution, and collecting missions of foreign countries have required complex permission procedures and conditions, if such permissions are granted at all [6]. As a result, the cross-border exchange of PGRFA has been severely and increasingly hindered, with obvious consequences for the effectiveness of collaboration among genebanks [7].

Luckily for Europe, there is a well-established umbrella organization of European countries, the European Cooperative Programme for Plant Genetic Resources (ECPGR), which has aimed at the coordination of PGRFA conservation and use activities since the early 1980s [8]. ECPGR is one of the regional PGRFA networks that were considered by the International Board for Plant Genetic Resources (IBPGR) to be part of the global conservation system of the Food and Agricultural Organization of the United Nations (FAO) in the late 1970s [2]. It organizes various collaborative activities, largely through its twenty Crop Working Groups and three Thematic Working Groups, which have resulted in valuable outputs such as the earlier mentioned EURISCO, many crop descriptor lists and crop-specific quality standards, and provides a platform for the formulation of EC-funded collaborative project proposals, and the organization of training workshops, etc. However, it has limited funds (contributed by the member countries) and does not have sufficient political leverage to directly enforce a rationalization of the conservation system at the regional level. The question ‘How can we make conservation of PGRFA in Europe more efficient?’ has been asked occasionally (e.g., [9]), but the answers have never translated into a restructuring of the existing landscape, since national and institutional interests have prevailed over the possibility to take decisions from a regional interest point of view.

At the global level, other important developments have influenced the political thinking with respect to the management of PGRFA. Besides the already mentioned CBD, the FAO launched the Global Plan of Action (GPA) for the Conservation and Sustainable Utilization of Plant Genetic Resources for Food and Agriculture in 1996 [10]. The GPA, updated in 2011 with the adoption of the second GPA [11], called for a more rational conservation system based on better planning and more collaboration and coordination, while allowing individual countries to maintain their sovereign rights over PGRFA. However, this had very little impact on the actual collaboration among countries, if any. Additionally, the establishment of the International Treaty on Plant Genetic Resources for Food and Agriculture (International Treaty) and its Multilateral System (MLS) for the access to and benefit-sharing of PGRFA in 2004 had little, or possibly even negative, impact on actual collaboration in Europe [7].

With regards to the above-described background, members of the ECPGR Steering Committee strongly felt that something had to fundamentally change, and subsequently formulated an idea that potentially could improve the existing situation: AEGIS, ‘A European Genebank Integrated System’ ([12], see also Box 1).

## 2. The Concept of AEGIS

Obviously, if Europe were to start from scratch and could operate as a unity, for instance, a system similar to that of the USDA National Plant Germplasm System [13] could be set up, i.e., including some central and some sub-regionally specialized facilities, with proper quality management and a clear policy regarding access. However, despite the potentially much larger cost-effectiveness of such an optimized and partly centralized system, its establishment is not conceivable, owing to the lack of political unity among the countries of the region, and the lack of strategic support from the European Union. Therefore, AEGIS had to be formulated in such a way that it could be implemented by the current actors, with the consequence that changes would have as little impact on the current activities as possible. ECPGR provided the political and administrative framework needed for the initiative, thus taking advantage of the existing common legal framework established by the International Treaty, as described above.

The concept of AEGIS was formulated in 2004 and resulted in a Policy Guide on AEGIS, endorsed by the ECPGR Steering Committee [14]. AEGIS aims at establishing a European Collection that is maintained in a decentralized virtual genebank consisting of various genebanks scattered over Europe and adhering to the AEGIS concept and principles. The European Collection consists of the collective set of accessions that have been identified and proposed by each country according to agreed criteria. Accessions of the European Collection are maintained for the long-term based on agreed quality standards in the decentralized virtual European genebank, i.e., the various participating genebanks, and are freely available under the terms and conditions set out in the International Treaty on Plant Genetic Resources for Food and Agriculture. A mechanism for the selection of the material to be included in this European Collection was created and eventually simplified [15], and the quality standards for maintenance [16] were defined.

The AEGIS concept was expected to result in a decentralized collection of unique and well-maintained accessions, assuring that the material in the European Collection would be safely conserved for the long term and freely accessible from the virtual European genebank. It was expected to give genebanks the option to drop the responsibility of maintaining accessions that were already well conserved in the European Collection by an identified colleague genebank. The benefits of AEGIS perceived at the time of its establishment are summarized in Table 2.

On 23 July 2009, upon signature of the Memorandum of Understanding by the tenth country eligible for membership, AEGIS entered into force. It took until 12 December 2011 for the first accessions to be included in AEGIS. However, retrospectively, there were a few flaws in the concept, and as a result it failed to become a full success in the first decade of its existence.

## 3. The Status of AEGIS

Currently, the European Collection consists of 65,267 accessions maintained by 46 genebanks. An overview of the current holdings in AEGIS is included in Table 3. This material was largely included because some individual genebanks submitted all the material in their collection that they considered as original material from their respective countries (i.e., collected by, or bred in the country), resulting in five genebanks submitting over three quarters of the total AEGIS accessions. Most of the additionally included accessions were part of joint crop-based and ECPGR-funded projects that required the inclusion of material in AEGIS. As a result, the content of AEGIS, about three percent of the accessions that are documented in EURISCO, is very much clustered around a few genebanks and around a few crops. This would be fine if all 65 thousand accessions in the European Collection were well-managed in accordance with the agreed criteria and met the AEGIS-agreed quality standards. However, this is not clear, and certainly not assured, as there is no operating auditing system.

Because of the slow growth of the European Collection, the ECPGR Steering Committee held an AEGIS review meeting in 2018 in Spain [17]. Its conclusions indicated that AEGIS could become more effective by (1) creating a network of ertified genebanks (with a sui generis certification system and definition of the standards); (2) creating a capacity building system of genebanks that want to become certified; (3) raising sufficient funding to support coordination, monitoring and capacity-building activities.

## 4. Critical Assessment of the Current Functioning of AEGIS

Considering the foregoing, and including the procedural steps that have been agreed upon by the AEGIS members, one should ask the question why AEGIS, which is such a good idea, has not (yet) become a success? Three elements can be distinguished: (1) too little material has been included for AEGIS to become ‘really operational’ and thus, to obtain benefits; (2) the quality of the genebanks’ operations has not been assured and is possibly too low in many cases; (3) trust in the continued availability of the accessions has not been assured, despite the formal inclusion of this aspect in the MOU and in the AEGIS Associate Membership Agreement. Let us, then, have a close and critical look at these elements.

### 4.1. Too Little Material Has Been Included in the European Collection

Why would a country/genebank include material in the European Collection? The public benefits of inclusion were spelled out in Table 2, and some high quality genebanks considered these sufficiently convincing arguments and designated accessions for inclusion in AEGIS. However, most countries and genebanks thought they were not or, at least, have not concluded an agreement with ECPGR or designated materials.

Obviously, there are several reasons to be reluctant to submit material. First of all, a country must commit itself (through the MOU) to conserve the designated accessions for the long term and to make them available to users. Furthermore, the genebank commits itself (through the Associate Membership Agreement) to meeting the quality standards (although no auditing system is implemented yet) to conserve the material ‘in perpetuity’ and provide complete access to the material under the SMTA. Thus, noting these obligations, and consequences such as making long-term commitments, accepting additional workloads and possibly requiring more funds to meet the requirements, one could ask ‘Why should they include materials? It only makes their lives difficult!’.

The reasons for joining AEGIS from a genebank point of view are supposed to be related to the advantages generated by membership of a cooperative framework: recognition of quality and the possibility of benefiting from common resources, such as the auditing system, capacity-building opportunities and sharing responsibilities among each other. Apparently, these advantages have not been sufficiently convincing. The label of being an ‘AEGIS genebank’ has not generated sufficient status yet, and access to the common resources has not materialized yet, e.g., participation in ECPGR-organized capacity-building activities is not dependent on AEGIS membership.

Furthermore, to reduce avoidable redundancy in the European Collection, the procedures for including material, i.e., individual accessions, were initially very complicated. However, these have been simplified, and the only remaining requirement is that the accession is ‘original’, meaning that it should be either unique, from a European perspective, to the holding genebank and/or that it originated through collecting or breeding from the country where the genebank is located.

Possibly, a reluctance to include material in the European Collection also exists at the national level. The AEGIS concept aims at generating a stable commitment from each country to conserve accessions of the European Collection for the long term. Countries appear very cautious, suggesting that their budgets for long-term conservation have not been secured and indicating that they cannot commit to the obligations formulated in the MOU [19].

The limited participation can also be interpreted as an indication that the perceived benefits of AEGIS for policymakers have not been fully appreciated. These benefits range from the assured compliance with International Treaty/the Nagoya Protocol for all the accessions included in AEGIS, to the fact that AEGIS offers a mechanism to optimize the use of resources by avoiding redundancy and assuring quality. In addition, AEGIS could strengthen the position of the European region in international fora, by offering an example of efficiency and commitment.

The fact that the above-mentioned benefits have not been able to facilitate the rapid growth and implementation of the AEGIS concept probably reflects a more general situation whereby the advantages of cooperating as a region rather than in isolation do not spontaneously emerge. This is especially understandable when considering the short-sighted political attitudes of many countries or segments of society within and outside the EU, which have been increasingly promoting nationalistic approaches for political agendas in the past ten years or so. This situation is somewhat surprising, as one would expect that, in postwar Europe, the extension of the European Union to include 27 countries, the peculiarities of PGRFA and the greatly varying state of development with respect to the conservation and sustainable use of PGRFA among European countries would call for intensified collaboration and coordination. In particular, the sharing of conservation and facilitating the use of PGRFA responsibilities among countries was thought to be a ‘given’ and as such, was included as a motivating factor for joining and operating AEGIS. However, this assumption seems to have some flaws, as the actual preparedness to share responsibilities among countries remains rather limited.

A few changes could be considered to improve this situation. First, a genebank should have a clear incentive to submit material to the European collection. This incentive is currently lacking, as the designation of accessions does not lower the operational costs of a genebank and does not bring other evident advantages to the genebank; to date, no duplicate accessions have been reported to have been eliminated from a genebank on the basis of AEGIS (one heard argument is that reduced numbers of accessions in a genebank could lead to a reduction in the institutional budget). Possibly, the assumption that individual genebanks would be eager to eliminate redundancies with other genebanks was incorrect. Moving from the ‘local genebank’ level to the national level, the argument might hold that conservation costs can be reduced, but this appears to be a non-incentive to genebanks without strong political support from the national government.

To establish an incentivizing framework, there might be a need to distinguish among genebanks that are able and willing to follow the agreed FAO Genebank Standards [20] and those that are not. In fact, one could argue that many of the 400 European genebanks or collections/repositories do not properly respond to a working definition of ‘genebank’, i.e., an institution that meets the requirements for well-managed and effectively operated collections. To distinguish these from ‘real genebanks’, an AEGIS certification system could be set up, establishing a European circle of AEGIS-certified genebanks to be supported at national and regional levels. Institutions that want to become AEGIS-certified genebanks, but do not meet the requirements yet, would need to be supported by ECPGR and other donors (e.g., the respective governments and the European Union) to reach this goal by capacity building, staff exchanges, support for setting up the required facilities, etc. In fact, it can be argued that the authorities concerned should demand from the genebanks they fund that they should become AEGIS certified and thus become amenable for eventual funding from European Union sources. ECPGR could obviously play an important task in setting up such a system, by creating the certification system and promoting the actual certification of genebanks through its Steering Committee. At the same time, more efforts are possibly required by ECPGR to make a strong case at the European level for such targeted funding. For instance, ECPGR is involved in the EU-funded Genres Bridge project, which has proposed a European Genetic Resources Strategy along the lines described here [21].

Once a critical mass of AEGIS-certified genebanks, i.e., the ‘real genebanks’, are identified, the inclusion of materials in the European Collection should become much more straightforward. Everything in a real genebank can be included, irrespective of its originality or other criteria. Subsequently, duplicated accessions in the European Collection would be able to be removed from the genebanks involved, as they would be available elsewhere in the European Collection, as indicated in EURISCO.

### 4.2. The Quality of Genebank Operations Has Not Been Assured

According to the AEGIS concept, if accessions are included in the European Collection, these accessions have to be properly managed, to such an extent that another genebank maintaining the same material can stop doing so. Obviously, proper genebank management requires assurances of the quality of operations of the participating genebanks. This implies that the genebanks (1) operate a quality management system; (2) meet the agreed quality standards; (3) are audited regularly. Obviously, these points would fit perfectly in an AEGIS certification system, as described under the previous point.

AEGIS has already made a good start in developing the standards by designing a quality management system called AQUAS [16], based on the FAO Genebank Standards [20]. The standard operating procedures proposed in these standards are based on realistic but sound quality levels, assuring the proper conservation of, and full access to the material in the European Collection. An outline of the required auditing system has also already been formulated [22], consisting of record keeping, reporting and monitoring steps. However, this has never been implemented, because the low number of AEGIS accessions has not yet justified the launching of a fully fledged auditing system. Another, possibly more important reason for not implementing the auditing system is the fact that the participating genebanks have been reluctant to introduce a monitoring system that could create an unwanted bureaucratic and reporting burden for the genebanks, as well as which could interfere with national or institutional management routines and decisions. Obviously, these considerations are completely contrary to current quality management concepts, which require the proper and transparent documentation of procedures, standardization where possible, and the monitoring of processes with appropriate performance indicators.

As a possible remedial action, to increase the transparency of European genebanks and to boost awareness of the importance of quality management, a genebank peer review process was created and successfully tested in 2019. It involves the documentation of the genebank processes (using AQUAS formats), mutual visits of experts to the genebanks involved, who provide frank and clear comments in a fully transparent way to each other, and reports about these visits [23].

### 4.3. The Continuity of the Availability of Accessions Has Not Been Assured

Genebanks can not and will not rely on each other if they cannot be sure about the continuity of the collections. History has shown, for instance, that genebanks can disappear, institutional policies can change, crop priorities within the national or institutional context are dynamic and evolve, and that national authorities can decide that germplasm can no longer leave the country without their consent. This obviously makes creating a collaborative system like AEGIS very difficult, and these are important points to be included in the formal agreements.

The MOU between ECPGR and the countries includes precautionary measures with respect to the withdrawal of accessions from the European Collection; a 12 months’ notice by the holding genebank is stipulated [19]. In case an associated genebank withdraws, a 12 months’ notice is also required, and in case a country wants to terminate the MOU, a 12 months’ notice is also required. These terms should give colleague genebanks the opportunity to request and receive the accessions to be withdrawn from the European Collection and include these in another AEGIS-certified genebank. However, the enforcement of the MOU has its limits, and recent history has shown that if a country doesn’t want to provide access to information or material, there is very little one can do to get access, irrespective of signed MOUs.

To overcome the risk of losing access to material from the European Collection, there could be an easy solution: if a genebank currently includes accessions that are conserved as seeds in the European Collection, it guarantees the availability of these materials to the rest of the world, and those accessions also have to be safety backed-up in another European genebank (or at the Svalbard Global Seed Vault). This is usually done in a ‘black-box’ arrangement, i.e., the holding genebank sends a sample of each accession to a colleague genebank that stores it under optimal conditions; this deposit will not affect any property or other rights on the material; the duplicated materials will remain in sealed containers; the terms and conditions governing the deposit will be agreed on between the two genebanks involved; the receiving genebank will not take any actions to further transfer the material other than back to the originator of the duplicated accessions or in accordance with the depositing genebank’s instructions, i.e., the material can only be retrieved from the genebank acting as the back-up location by the genebank which sent it there ([19], art. 1-iv). No one else has access. For PGR accessions that are not conserved as seed, alternative solutions are sought, but on the same principles. This safety backup ‘system’ could easily be modified to serve as an instrument to guarantee continuity of access. This would be guaranteed if the safety duplication is done under the provision that the accessions in the back-up genebank can be used for inclusion in another genebank collection in the undesirable case that the original holding genebank could no longer provide access to these accessions. This simple change would assure that the material stays within the AEGIS system and remains available even if an institute or country is not able to comply with the availability clauses of the MOU anymore, or even decides to withdraw its membership. The material could simply be reintroduced into the European Collection by another AEGIS-certified genebank. The rationale for proposing this change lies in the recognition that AEGIS-designated materials are treated as part of the MLS and, therefore, after their first exchange with an SMTA, they can be indefinitely transferred under the same conditions to any user. Therefore, it does not make sense to maintain the obligation to return the material only to the original depositor such as in most ‘black-box’ arrangements for safety duplication.

## 5. Future of AEGIS

A reasonable response to the points above would be “Dream on!”, and, indeed, the proposed solutions to the points above will not be easy to implement. A genebank certification system might be very difficult to set up, as some countries will fear that they would never be able to meet the standards and thus prefer to avoid confrontation with the reality that some genebanks do not live up to the level of quality that is expected from public goods institutions operating in the global arena. Furthermore, establishing and running an auditing system requires an adequate budget (setting up a quality management system could cost up to 10% of an annual budget, and operating it up to 5% of the annual budget, but could be much less) and such funds are currently lacking. Therefore, setting up an auditing system without sufficient support or proper funding will be very difficult. Changing the safety back-up system from the current black-box construction to an emergency-access system might be considered undesirable, since it could discourage some genebanks from backing up their material for fear of losing control.

Nevertheless, some suggested steps can be made to improve the situation, paving the way for an easier and more comprehensive implementation of AEGIS and its European Collection. The European genebank community clearly has the desire to professionalize, to move from the first generation of genebank managers to the second. The already mentioned quality management system AQUAS provides valuable tools, such as the Genebank Manual [24], allowing genebanks to describe their current procedures, which is a first step towards proper quality management and a great way to improve transparency. Implemented with the hope of increasing transparency and of moving towards an auditing system, the peer reviews appeared successful, and several genebanks volunteered to participate. The first experiences with this approach have been very positive [23].

Furthermore, other initiatives to improve transparency and create a clearer picture about the quality of the European genebanks can and should be taken. For example, checking the availability and quality of the material in the European Collection is easy; one can simply request the material from the genebank and check its quality. To avoid wasting materials in this process, such requests should be done together with users that actually will use the material. A first attempt to create such a ‘system’ was undertaken by the Centre for Genetic Resources, The Netherlands (CGN), in 2019, when this genebank asked its users to draft a list of materials from other European genebanks that they would like to receive for their use. CGN requested these materials from these genebanks with full transparency about the context of these requests. Most of the requested material was not received. However, the COVID-19 crisis could be the main explanation for the (temporary?) lack of access that was experienced. The ECPGR Executive Committee was informed accordingly about this experiment, and it was favorable to extending it in scope, in terms of crops, genebanks and breeding community [25].

The above-mentioned steps can improve the situation in the European genebank community and reduce its rather uncoordinated and unharmonized aspects. However, they remain small steps, to be taken slowly. Apparently, national and institutional interests are currently still larger than concerns about an efficient PGRFA conservation system in Europe. All we can do is to continue trying to make small steps in the right direction. The initiatives developed in the framework of the EU-funded Genres Bridge project might have a very positive effect, provided that funds will become available. In particular, the European Genetic Resources Strategy, recently drafted by the GenRes Bridge partners, calls for the establishment of a coherent policy framework for genetic resources in Europe, facilitating and promoting genetic resources conservation, documentation and sustainable use at both national and European levels [21]. It also calls for the further development of a European infrastructure for ex situ and in situ PGRFA conservation and sustainable use. This infrastructure should include, inter alia, the decentralized/virtual European Genebank consisting of certified genebanks, building on the AEGIS experience and principles.

## 6. Conclusions

The world is not a perfect place, and the European PGRFA activities are no exception. The chaotic, casually grown European landscape of PGRFA actors and activities, although it does a lot of good, is far from ideal. The optimal solution is not feasible, given the current funding and decision-making mechanisms. ECPGR made an excellent attempt to improve the situation with AEGIS; however, this has not really worked so far: the impact on efficacy and efficiency has been very limited.

The possibility of reducing costs by reducing redundancy appears to have very limited appeal to most countries or genebanks. The concept of ‘national sovereignty’ over PGRFA, promoted by the CBD, appears to have a stronger appeal than the advantages of making PGRFA a common good in Europe. Therefore, implementing AEGIS will remain a significant challenge, as long as genebanks are funded by national authorities. Having additional EU regional funding would make the challenge of creating an effective and efficient European genebank infrastructure much easier.

With additional funding, a system of AEGIS-certified genebanks could be set up, in which the quality and continuity of conservation of, and access to PGRFA could be guaranteed. Joining this system would be attractive as it would certify that a genebank is a ‘real genebank’, with reliable conservation, access and legal protocols. Regional funding aimed at ex situ PGRFA management should concentrate on these certified genebanks, helping them to make the large steps that are needed to function optimally in a rapidly changing, increasingly -omics, research and breeding oriented environment. Obviously, a capacity-building program supporting genebanks that want to reach certification should be a prominent part of this vision. In the meanwhile, attempts to make small steps towards the goals of AEGIS are ongoing.

A decentralized, ‘virtual’ European genebank is a valid model, but apparently very hard to implement without proper regional and political visions, funding and decision making.

## Figures and Tables

**Table 1:** Salient statistical features of information in EURISCO.

	Number	Percentage of Acc’s in EURISCO
Number of accessions	2,056,983	
part of MLS:	430,597	20.90%
part of AEGIS:	65,286	3.20%
with a DOI:	228,078	1.10%
Number of institutes:	401	
Number of countries:	43	
Number of genera:	6725	
Number of species:	45,179	

Source: [3].

**Table 2:** Perceived public benefits of AEGIS.

(a)	Improved collaboration among European countries.
(b)	Cost-efficient conservation activities within and among European genebanks.
(c)	Reduced redundancy in European collections.
(d)	Improvement of quality standards for conservation, information management and the facilitation of the use of conserved germplasm across Europe.
(e)	More effective and better quality regeneration.
(f)	Facilitated access to all the germplasm included in AEGIS.
(g)	Improved security of germplasm through standardized commitments and safety duplication.
(h)	Improved linkages between ex situ and in situ conservation as well as linkages with users.
(i)	Improved sharing of knowledge and information.

Source: [14].

**Table 3:** Overview of accessions in AEGIS.

Total number of AEGIS accessions (included in the European Collection)	65,267	
Number of countries with AEGIS accessions	19	Germany 26,725 acc.; Italy 16,336 acc.; Netherlands 5841 acc.; Switzerland 5611 acc.; Nordic Countries 4785 acc.; 14 other countries 5969 acc.
Number of genebanks with AEGIS accessions	46	
Number of AEGIS genera	366	*Hordeum* 15,667 acc.; *Triticum* 11,129 acc.; *Zea* 5699 acc.; *Lolium* 2727 acc.;
		*Solanum* 2449 acc.; *Pisum* 2341 acc.; *Brassica* 2289 acc.; *Festuca* 2256 acc.;
		other 358 genera 20,710 acc.

Source: [18].

## Abbreviations and Acronyms

AEGIS	A European Genebank Integrated System
AQUAS	AEGIS Quality System
CBD	Convention on Biological Diversity
CGIAR	originally: Consultative Group on International Agricultural Research, now used as acronym-name
CGN	Centre for Genetic Resources, The Netherlands
ECPGR	European Cooperative Programme for Plant Genetic Resources
EURISCO	European Search Catalogue for Plant Genetic Resources
FAO	Food and Agriculture Organization of the United Nations
GPA	Global Plan of Action for Plant Genetic Resources for Food and Agriculture
IBPGR	International Board for Plant Genetic Resources
Treaty	International Treaty on Plant Genetic Resources for Food and Agriculture (FAO)
MLS	Multilateral System of Access and Benefit-sharing
MOU	Memorandum of Understanding
PGRFA	Plant Genetic Resources for Food and Agriculture
SMTA	Standard Material Transfer Agreement
